# Plant Community Diversity Influences Allocation to Direct Chemical Defence in *Plantago lanceolata*

**DOI:** 10.1371/journal.pone.0028055

**Published:** 2011-12-09

**Authors:** Anne Mraja, Sybille B. Unsicker, Michael Reichelt, Jonathan Gershenzon, Christiane Roscher

**Affiliations:** 1 Max Planck Institute for Chemical Ecology, Jena, Germany; 2 Max Planck Institute for Biogeochemistry, Jena, Germany; University of Zurich, Switzerland

## Abstract

**Background:**

Forecasting the consequences of accelerating rates of changes in biodiversity for ecosystem functioning requires a mechanistic understanding of the relationships between the structure of biological communities and variation in plant functional characteristics. So far, experimental data of how plant species diversity influences the investment of individual plants in direct chemical defences against herbivores and pathogens is lacking.

**Methodology/Principal Findings:**

We used *Plantago lanceolata* as a model species in experimental grasslands differing in species richness and composition (Jena Experiment) to investigate foliar concentrations of the iridoid glycosides (IG), catalpol and its biosynthetic precursor aucubin. Total IG and aucubin concentrations decreased, while catalpol concentrations increased with increasing plant diversity in terms of species or functional group richness. Negative plant diversity effects on total IG and aucubin concentrations correlated with increasing specific leaf area of *P. lanceolata*, suggesting that greater allocation to light acquisition reduced the investment into these carbon-based defence components. In contrast, increasing leaf nitrogen concentrations best explained increasing concentrations of the biosynthetically more advanced IG, catalpol. Observed levels of leaf damage explained a significant proportion of variation in total IG and aucubin concentrations, but did not account for variance in catalpol concentrations.

**Conclusions/Significance:**

Our results clearly show that plants growing in communities of varying species richness and composition differ in their defensive chemistry, which may modulate plant susceptibility to enemy attack and consequently their interactions with higher trophic level organisms.

## Introduction

In the last decades, the important role of plant diversity for ecosystem functioning has been verified in numerous studies [1], [2]. Increasing productivity is one of the well documented positive effects of plant species richness in plant communities (e.g. [3], [4], [5]). To what extent plant species richness affects the performance of single plant species has not been as intensively studied but there are examples in the literature documenting positive, neutral and negative effects [6], [7], [8]. Apart from simple increases in plant species richness, other changes in community composition and the presence or absence of certain plant functional groups, particularly legumes and grasses, strongly impact plant performance (e.g. [9], [10]).

Changes in plant diversity drastically affect both the biotic and abiotic environment of individual plant species. Increasing plant diversity correlates with increasing canopy density and height [11], [12] as well as soil nutrient exploitation [13]. The presence of particular species or plant functional groups such as nitrogen-fixing legumes additionally affects neighboring plants through direct or indirect provision of symbiotically fixed atmospheric nitrogen or reduced competition for soil nitrogen [14], [15]. The load and impact of plant natural enemies such as invertebrate herbivores or fungal pathogens also vary along a plant diversity gradient. Herbivore loads and levels of invertebrate herbivory have been shown to be influenced either positively or negatively by increasing plant diversity [16], [17]. Thus, the characteristics of a plants ecological niche may vary significantly along a plant diversity gradient. The fate of an individual plant strongly depends on its ability to respond plastically to the environment. As suggested by the optimal resource allocation theory [18], [19], plants that face a limited pool of resources should adjust their allocation and invest a higher proportion of resources in organs that capture the most limiting resource. However, the multiple changes in the individual plants environment that occur along a diversity gradient require a coordinated response to achieve the appropriate balance among different functions.

Several hypotheses have been put forward to explain plant resource allocation to defence metabolites and structures active against natural enemies (see review [20]). Selective factors shaping plants allocation into chemical defence are the value of plant tissue to the plant, the benefit of defence and the probability of enemy attack [20], [21]. Plant-plant- and plant-herbivore-interactions are both important biotic factors well known to modify plant chemical defences. The strength and shape of these biotic interactions also change with increasing plant diversity and with plant species composition. Thus it is obvious to address the question, whether plant diversity itself can directly or indirectly modify plant defence properties. To our knowledge this question has not been investigated so far. The aim of our study was to investigate effects of plant diversity on patterns of plant allocation to direct defence. Our model species was the perennial forb *Plantago lanceolata* L. (Plantaginaceae), a cosmopolitan species native to European grasslands. The major low molecular weight defence compounds in *P. lanceolata* are the monoterpene-derived iridoid glycosides (IG), aucubin and catalpol, which are repellent and/or toxic especially against generalist insect herbivores [22]. In *P. lanceolata*, iridoid glycoside production is heritable [23] and may vary considerable among adult plants [24], [25]. Iridoid glycoside concentrations have been shown to change during ontogeny [25], [26] and in response to variation in nutrient availability and herbivory [27], [28]. We used experimental grassland communities (Jena Experiment, [29]) that vary both in the number of plant species (from 1–60) and in the number and identity of plant functional groups (1–4 functional groups, including legumes, small herbs, tall herbs, and grasses). We harvested shoots of *P. lanceolata*, determined foliar iridoid glycoside concentrations, assessed herbivore damage and measured leaf ecophysiological characteristics (specific leaf area, foliar nitrogen concentrations) known to provide valuable information about species adjustment to changes in light and nutrient availability (for review see [30]). We tested the following hypotheses: 1) Changes in plant species richness and plant community composition impact foliar concentrations of defensive metabolites. 2) Plant diversity effects on defensive plant metabolites correlate with changes in light and nitrogen availability and the intensity of herbivore damage. 3) Irrespective of changes in plant diversity, young leaves and reproducing plant shoots being organs of higher value have higher concentrations of defence metabolites than old leaves and vegetative shoots.

## Results

### Diversity effects on foliar iridoid glycoside concentrations

**Figure 1:**
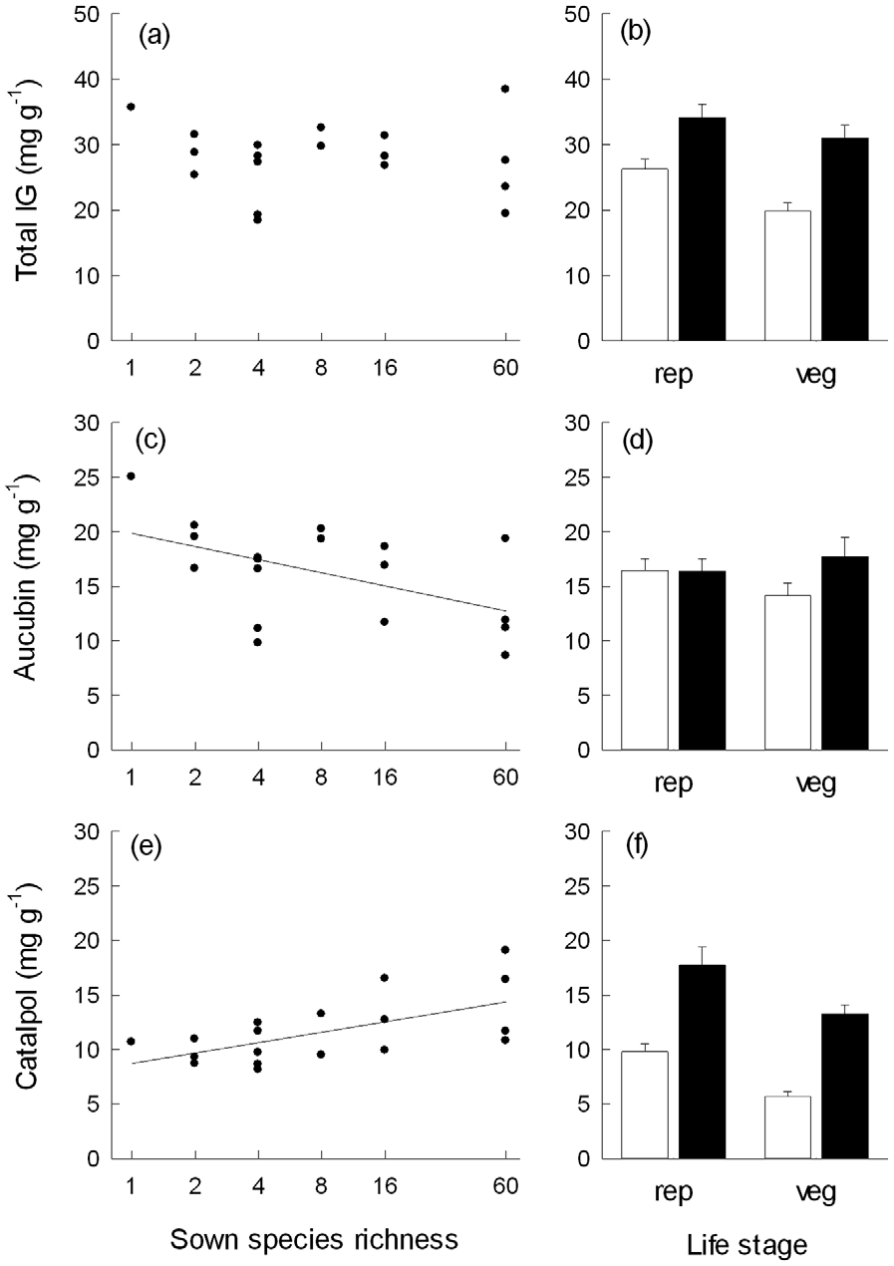
Relationships between species richness and total iridoid glycoside, aucubin and catalpol concentrations in leaves of *Plantago lanceolata.* Total iridoid glycoside concentrations (a–b), aucubin concentrations (c–d), and catalpol concentrations (e–f) in leaves of *P. lanceolata* plotted as mean values per plot against sown species richness (a, c, e) and as mean values (±1 SE) for older and younger adult leaves of reproductive (rep) and vegetative (veg) shoots across plots (b, d, f). Regression lines indicate significant species-richness effects.

Total foliar iridoid glycoside (IG) concentrations did not vary in response to increasing species richness (Fig. 1a), although a significant interaction “Life stage×SR” (Table 1) indicated that species-richness effects on total IG concentrations depended on life stage. However, separate analyses for vegetative shoots (L = 3.12, p = 0.078) and reproductive shoots (L = 0.94, p = 0.331) did not result in significant species-richness effects for both life stages. Total IG concentrations decreased with increasing numbers of plant functional groups present in the experimental communities. The presence/absence of particular functional groups, namely legumes or grasses did not affect total IG concentrations, although a significant interaction “Life stage×Leg” suggested that variation in total IG concentrations in response to legume presence depended on life stage (Table 1). Among the two iridoids measured, aucubin concentrations decreased and catalpol concentrations increased with increasing species richness (Fig. 1c, e; Table 1). While aucubin concentrations declined by near 50%, the increase in catalpol concentrations amounted to 35% from the *P. lanceolata* monoculture to the 60-species mixture resulting in a decrease of aucubin∶catalpol ratios from about 2.5 to 1.2. Separate analyses showed that only leaves of reproductive shoots had significantly higher catalpol concentrations at increasing species richness (L = 10.44, p = 0.001), while species-richness effects were non-significant for vegetative shoots (L = 0.16, p = 0.691). Species richness effects on aucubin concentrations did not depend on life stage (Table 1). An increasing number of plant functional groups in the communities had additional positive effects on catalpol concentrations. Significant interactions between life stage and the presence/absence of grasses and legumes respectively indicated that effects of functional group composition on foliar aucubin concentrations depended on life stage (Table 1). The presence of grasses increased foliar aucubin concentrations of reproductive shoots (L = 5.75, p = 0.017), while legume presence tended to decrease their foliar aucubin concentrations (L = 3.14, p = 0.076). In contrast, the presence of grasses (L = 0.06, p = 0.804) or legumes (L = 0.26, p = 0.611) did not affect foliar aucubin concentrations of vegetative shoots.

**Table 1:** Summary of mixed-effects model analyses of total iridoid glycoside, aucubin and catalpol concentrations.

	Total IG concentration			Aucubin concentration			Catalpol concentration		
	L ratio	p value		L ratio	p value		L ratio	p value	
Species number (SR)	0.16	0.688		5.61	0.018	↓	8.06	0.005	↑
Functional group number (FG)	6.00	0.014	↓	3.44	0.064		4.87	0.027	↑
Legume presence (LEG)	0.03	0.865		0.38	0.535		1.92	0.166	
Grass presence (GR)	0.01	0.906		1.23	0.268		2.55	0.110	
Life stage	10.65	0.001	R	0.28	0.597		20.49	<0.001	R
SR × Life stage	8.55	0.003		2.41	0.120		9.54	0.002	
FG × Life stage	1.60	0.206		0.32	0.574		2.02	0.156	
LEG × Life stage	5.50	0.019		5.31	0.021		2.08	0.150	
GR × Life stage	3.81	0.051		5.79	0.016		0.55	0.457	
Leaf age	111.81	<0.001	Y	10.94	0.001	Y	133.66	<0.001	Y
Leaf age × Life stage	4.43	0.035		12.54	<0.001		0.11	0.736	

Models were fitted by stepwise inclusion of terms. Listed are the results of likelihood ratio tests that were applied to assess model improvement ( = L ratio) and the statistical significance of the explanatory terms ( = p values). Note that contrasts for the presence/absence of legumes and grasses were entered separately in a series of analyses. Arrows indicate a significant increase (↑) or decrease (↓) of variables in relation to the experimental factors of the biodiversity experiment, R indicates higher values in reproductive shoots, and Y indicates higher values in the younger leaf cohort.

In general, leaves of reproductive shoots had higher total IG and catalpol concentrations. Total IG, catalpol and aucubin concentrations were higher in younger than in older adult leaves (Figs. 1b, d, e; Table 1).

### Diversity effects on leaf damage, light and nitrogen acquisition

**Figure 2:**
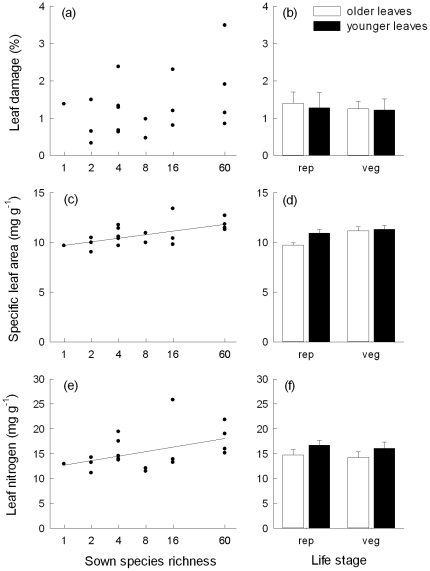
Relationships between species richness and proportional leaf damage, leaf nitrogen concentrations and specific leaf area of *Plantago lanceolata*. Proportional leaf damage (a–b), leaf nitrogen concentrations (c–d), and specific leaf area (e–f) of *P. lanceolata* plotted as mean values per plot against sown species richness (a, c, e) and as mean values (±1 SE) for older and younger adult leaves of reproductive (rep) and vegetative (veg) shoots across plots (b, d, f). Regression lines indicate significant species-richness effects.

The proportion of leaf damage varied independently from plant diversity (Fig. 2a), and did not depend on life stage (Table 2). Leaf damage was higher in older than in younger adult leaves (Fig. 2b; Table 2). Leaf damage correlated positively with specific leaf area (SLA; r = 0.49, p = 0.038, N = 18) and leaf nitrogen concentrations (N_M_; r = 0.73, p = 0.001) at the plot-level. SLA increased with increasing species richness (Fig. 2c), but functional group number or the presence of particular functional groups did not influence SLA (Table 2). SLA averaged across older and younger adult leaves of reproductive and vegetative shoots correlated positively with community leaf area index (LAI; r = 0.679, p = 0.002, N = 18) and canopy height of the surrounding vegetation (r = 0.713, p = 0.001). SLA was higher in leaves of vegetative shoots and in the younger leaf cohort (Fig. 2d; Table 2). N_M_ was positively related to increasing species richness (Fig. 2e; Table 2). N_M_ was significantly lower when grasses were present and significantly higher when legumes were present. Leaves of reproductive shoots and the younger leaf cohort had higher N_M_ (Fig. 2f).

**Table 2:** Summary of mixed-effects model analyses of proportional leaf damage, specific leaf area and leaf nitrogen concentration.

	Leaf damage			Specific leaf area			Leaf nitrogen concentration		
	L ratio	p value		L ratio	p value		L ratio	p value	
Species number (SR)	0.23	0.629		7.36	0.007	↑	4.38	0.036	↑
Functional group number (FG)	1.73	0.189		2.10	0.148		0.11	0.740	
Legume presence (LEG)	0.81	0.368		1.06	0.302		6.43	0.011	↑
Grass presence (GR)	1.55	0.214		3.72	0.054		6.94	0.008	↓
Life stage	1.19	0.274		8.50	0.004	V	6.64	0.010	R
SR × Life stage	2.30	0.129		0.74	0.389		0.98	0.323	
FG × Life stage	0.41	0.521		0.09	0.769		0.28	0.600	
LEG × Life stage	0.35	0.552		0.03	0.868		2.31	0.129	
GR × Life stage	2.48	0.115		0.02	0.894		0.02	0.881	
Leaf age	5.46	0.020	O	14.78	<0.001	Y	78.36	<0.001	Y
Leaf age × Life stage	0.02	0.899		7.61	0.006		0.72	0.397	

Models were fitted by stepwise inclusion of terms. Listed are the results of likelihood ratio tests that were applied to assess model improvement ( = L ratio) and the statistical significance of the explanatory terms ( = p values). Note that contrasts for the presence/absence of legumes and grasses were entered separately in a series of analyses. Arrows indicate a significant increase (↑) or decrease (↓) of variables in relation to the experimental factors of the biodiversity experiment, R (reproductive shoots) and V (vegetative shoots) indicate higher values dependent on life stage, and O (older leaf cohort) and Y (younger leaf cohort) indicate higher values dependent on leaf age.

### Effects of resource availability and leaf damage on iridoid glycoside concentrations

**Figure 3:**
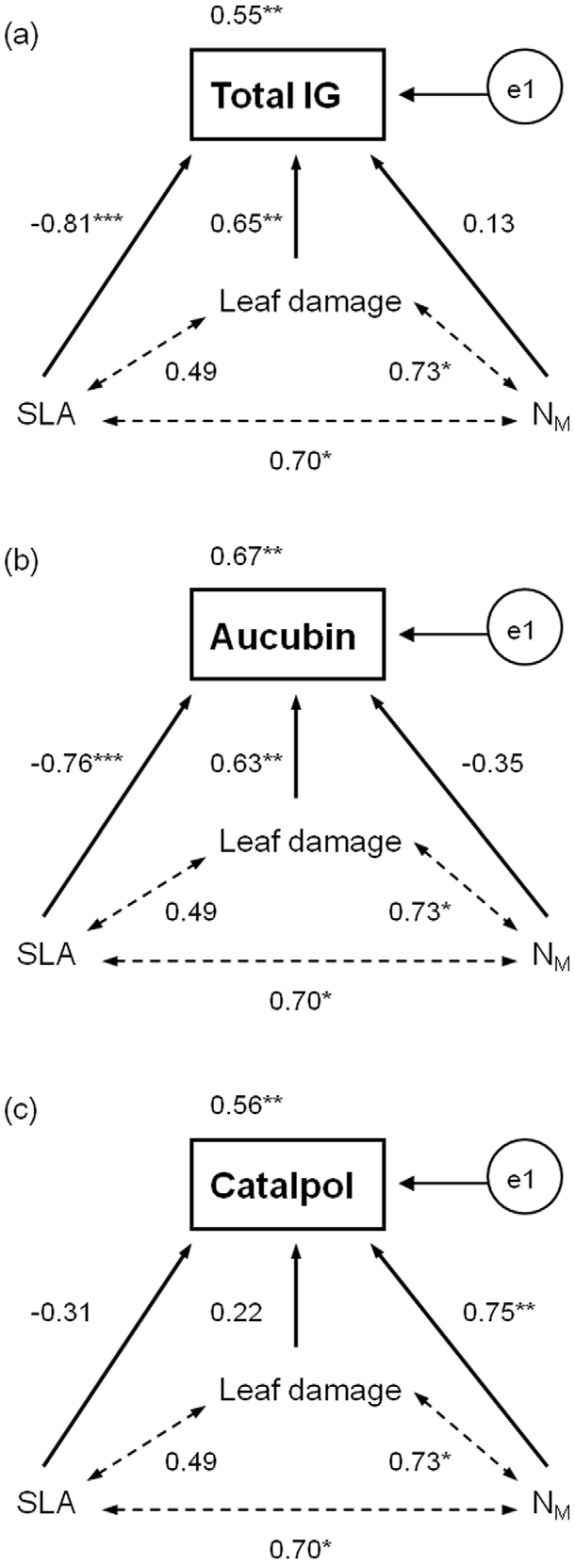
Path analysis of the relationships between iridoid glycoside concentrations and specific leaf area (SLA), leaf nitrogen concentrations (N_M_) and proportional leaf damage of *Plantago lanceolata*. Standardized path coefficients are given next to the path arrows (solid lines), dashed arrows indicate covariance. Unexplained variation is denoted with e1. Significance levels are * p≤0.05, ** p<0.01, *** p<0.001. Values are means across samples of two younger and older fully developed leaves of five vegetative and reproductive shoots of *P. lanceolata* per plot. Regression lines indicate significant species-richness effects.

Path analyses visualized significant covariances among SLA, N_M_ and leaf damage averaged at the plot level (Fig. 3). Total IG and aucubin concentrations correlated positively with levels of leaf damage and negatively with SLA (Fig. 3a, c). Catalpol concentrations were positively correlated with N_M_ (Fig. 3b).

Mixed-effects model analyses accounting for within-plot variation through differences between older and younger adult leaves of reproductive and vegetative shoots confirmed significant effects of resource availability and leaf damage on foliar concentrations of defence compounds. Leaf nitrogen concentrations and proportional leaf damage increased, and specific leaf area decreased total IG concentrations. However, negative effects of functional group number on total IG concentrations remained significant suggesting that plant diversity effects are not fully mediated by concomitant changes in resource availability and proportional leaf damage (Table 3). Variation in aucubin concentrations was partly attributable to SLA and leaf damage, while N_M_ had significant effects on catalpol concentrations (Table 3). Species richness and functional group number fitted after the combination of covariates had additional negative effects on aucubin concentrations, while species-richness effects on catalpol concentrations did not remain statistically significant after accounting for covariates (Table 3). Different total IG, aucubin and catalpol concentrations in older and younger adult leaves of reproductive and vegetative shoots were not explained by variation in N_M_, SLA or leaf damage.

**Table 3:** Summary of mixed-effects model analyses including a combination of measured covariates to assess effects resource availability and leaf damage by herbivores on total iridoid glycoside, aucubin and catalpol concentrations.

	Total IG concentration			Aucubin concentration			Catalpol concentration		
	L ratio	p value		L ratio	p value		L ratio	p value	
Covariate 1	Leaf nitrogen concentration			Specific leaf area			Leaf nitrogen concentration		
	6.34	0.012	↑	11.02	0.001	↓	29.99	<0.001	↑
Covariate 2	Leaf damage			Leaf damage			Leaf damage		
	4.08	0.044	↑	7.29	0.007	↑	1.11	0.291	
Covariate 3	Specific leaf area			Leaf nitrogen concentration			Specific leaf area		
	4.41	0.036	↓	0.52	0.472		0.69	0.407	
Species number (SR)	0.80	0.372		4.03	0.045	↓	2.99	0.084	
Functional group number (FG)	5.58	0.018	↓	3.92	0.048	↓	6.46	0.011	↑
Legume presence (LEG)	0.47	0.495		0.16	0.690		0.64	0.425	
Grass presence (GR)	1.00	0.317		0.73	0.393		0.69	0.405	
Life stage	7.89	0.005	R	0.04	0.847		16.91	<0.001	R
SR × Life stage	7.69	0.006		2.66	0.103		8.04	0.005	
FG × Life stage	1.73	0.188		0.25	0.617		2.32	0.128	
LEG × Life stage	5.92	0.015		5.75	0.017		2.33	0.127	
GR × Life stage	3.55	0.060		7.63	0.006		0.21	0.649	
Leaf age	108.28	<0.001	Y	20.68	<0.001	Y	116.72	<0.001	Y
Leaf age × Life stage	3.18	0.075		9.91	0.002		0.13	0.722	

Models were fitted by stepwise inclusion of terms. Order of covariates was determined through step-wise model extension starting with separate models for each covariate. Models were compared using Akaike's information criterion (AIC), and the model with the lowest AIC containing one of the covariates was selected. In further steps the model was extended until all covariates were fitted, and the experimental factors were entered in the same order as in models without additional covariates (see Table 1). Listed are the results of likelihood ratio tests that were applied to assess model improvement ( = L ratio) and the statistical significance of the explanatory terms ( = p values). Note that contrasts for the presence/absence of legumes and grasses were entered separately in a series of analyses. Arrows indicate a significant increase (↑) or decrease (↓) of variables in relation to measured covariates or the experimental factors of the biodiversity experiment, R indicates higher values in reproductive shoots, and Y indicates higher values in the younger leaf cohort.

### Diversity effects on performance of *P. lanceolata*

Density of *P. lanceolata* plants (corrected for sown proportions) increased with increasing species richness (L = 16.08, p<0.001; Fig. S1a), while functional group number did not affect plant density. In mixtures with grasses density of *P. lanceolata* was higher than in mixtures without grasses (L = 4.85, p = 0.028), while legume presence did not influence shoot density of *P. lanceolata*. Increasing species richness had positive effects on shoot biomass of *P. lanceolata* (L = 12.44, p<0.001; Fig. S1b). On average, shoot biomass was larger in communities with legumes (L = 5.74, p = 0.017), while the presence of grasses affected shoot biomass negatively (L = 6.66, p = 0.010). Reproductive shoots of *P. lanceolata* had a higher biomass than vegetative shoots (Fig. S1c), but effects of plant diversity on shoot biomass did not depend on life stage. Shoot biomass correlated positively with foliar catalpol concentrations averaged at the plot level (r = 0.53, p = 0.023, N = 18), while total IG and aucubin concentrations were not related to shoot biomass of *P. lanceolata*.

## Discussion

Our results clearly show that plant diversity affects the chemical defence of *P. lanceolata* by modifying the abiotic and biotic conditions of the plants' environment. Plant species richness did not influence total iridoid glycoside (IG) concentrations, but aucubin concentrations alone correlated negatively with plant species richness, and catalpol concentrations increased with plant species richness in the experimental grassland plots. In addition, an increasing number of plant functional groups was positively related to catalpol concentrations in *P. lanceolata* and had negative effects on total IG concentrations. Notably specific leaf area (SLA) and leaf damage explained most of the variation in aucubin concentrations, while most of the variation in catalpol concentrations was attributable to variation in leaf nitrogen concentrations (N_M_).

Increasing plant species richness has been shown to correlate positively with increasing canopy height and community leaf area index (LAI) [11], [12], [31], thus reducing light availability for subordinate plant species. The positive correlation between SLA and plant species richness (and LAI) indicated that *P. lanceolata* exhibits typical leaf structural adjustment to enhance light acquisition [32]. Increased leaf nitrogen concentrations of *P. lanceolata* in the presence of legumes indicated that these nitrogen-fixing plants improved the nitrogen supply of *P. lanceolata* individuals. In contrast, grasses, known to be very efficient in acquiring soil nitrogen [13], decreased total leaf nitrogen concentrations in plantain. In accordance with the results by [33] herbivore damage in *P. lanceolata* was not related to plant diversity. Although we did not find significant relationships between community LAI and herbivore damage [33], positive relationships between SLA and herbivore damage suggested that *P. lanceolata* was more heavily attacked by insect herbivores when growing deeper in the canopy.

Light and nutrient availability [27], [28], [34], plant neighbour identity [35] as well as increased competition at higher plant density [36] all have been shown to alter plant allocation patterns to chemical defence. Various theories (e.g., carbon-nutrient balance hypothesis [37], resource availability hypothesis [38], growth-differentiation balance hypothesis [39]) postulate that variation in plant resistance can partly be attributed to competition and resource availability. Additionally, the constitutive level of IG in *P. lanceolata* is known to increase upon induction in response to herbivores [28] or fungal pathogens [40]. In our study, lower total IG and aucubin concentrations at increasing species or functional group richness (Table 1) correlated with increasing SLA of *P. lanceolata* (Fig. 3a, c; Table 3). Therefore, it is likely that carbon limitation due to insufficient light availability associated with lower rates of photosynthesis caused a lower investment into carbon-based defence compounds at increasing plant diversity. In contrast, foliar concentrations of catalpol, which is more inducible by specialist herbivores and thus may be a better defence against them [25], increased with increasing plant diversity. Contrary to expectations based on previous studies that investigated the effects of nutrient availability on carbon-based allelochemicals [27], [41], we found a positive correlation between foliar catalpol and nitrogen concentrations although leaf nitrogen concentrations only partly explained variation in catalpol concentrations (Table 3). Because aucubin is the biosynthetic precursor of catalpol, increasing proportions of catalpol require a greater metabolic investment than aucubin [25]. Due to the fact that the total IG concentrations of *P. lanceolata* were only weakly related to leaf nitrogen concentrations, it is likely that the plant increased its defensive allocation to more nutritious nitrogen-rich leaves by investing energy into the synthesis of a more effective defence, catalpol.

Observed levels of herbivore leaf damage only weakly explained variation in total IG concentrations (Table 3). Nevertheless, we cannot disentangle the importance of herbivore induced production of defence-compounds in our experiment. Visible leaf damage is only a rough estimate of herbivore effects on plants [42], as it only allows estimates for leaf chewing herbivore damage. Furthermore, timing of herbivore damage may be crucial for patterns and dynamics of IG induction but based on the data from our experiment, we have no information about the actual incidence of herbivore attack. Laboratory experiments have yielded conflicting results on induction of iridoid glycoside concentrations through herbivory. Herbivory on *P. lanceolata* has been shown to result in higher catalpol concentrations and catalpol∶total IG ratios than in undamaged plants suggesting that catalpol is induced in response to herbivory [25]. In our study leaf nitrogen concentrations were positively correlated with leaf damage; therefore higher levels of the more toxic catalpol could also be induced by herbivore attack.

Fitness benefits of defence compounds that potentially reduce the level of pathogen infestation and herbivore attack may be counterbalanced by their costs [43], [44]. Allocation costs occur as trade-off between the investment of limited resources into resistance-related traits and other fitness-related traits. Ecological costs arise when increased resistance against certain enemies impairs resistance against other enemies, plants competitive ability or efficacy of beneficial organisms such as mycorrhizae or pollinators [43]. The biosynthesis of monoterpene-derived iridoid glycosides is likely associated with high metabolic costs for the plant [45]. In our experimental communities shoot biomass and density (corrected for sown proportions) as indicators for species performance increased with plant diversity, and legumes had additional positive effects on shoot biomass. Total IG and aucubin concentrations were not related to shoot biomass of *P. lanceolata*, while catalpol concentrations correlated positively with shoot biomass. Changes in plant diversity may also change the prevalence of different herbivore types. For example, the increased proportion of catalpol in plants with increasing species richness may be a response to the increased likelihood of attack by specialist herbivores in richer plant communities, rather than generalist herbivores, since catalpol has been shown to be more induced by specialist herbivory than aucubin [25].

The optimal defence hypothesis [21] is based on the postulation that defence compounds are costly to produce. Recent meta-analysis [46] has shown that plant organs with higher assumed value, such as younger leaves compared to older leaves, had higher concentrations of chemical defence compounds, while the magnitude of differences was less pronounced when reproductive parts (higher assumed value) were compared to leaves (lower assumed value). The results from our study strongly support the optimal defence hypothesis as we measured significantly higher levels of total IG and catalpol concentrations in leaves of reproductive shoots than in vegetative shoots. Furthermore iridoid glycoside concentrations were higher in younger than in older adult leaves. Similar results were obtained in previous laboratory experiments [41], [47]. These differences among leaf cohorts and vegetative vs. reproductive shoots were near-independent of plant diversity.

Previous studies have shown that iridoid glycoside production in *P. lanceolata* is heritable [23] and may differ significantly among genotypes [25], [27]. The experimental plots of the Jena Experiment were all established from the same seed population purchased from a commercial supplier (for details see [29]). A reciprocal transplant-replant experiment with offspring from seed families of *P. lanceolata* collected five years after sowing in monoculture and 60-species-mixture showed that variation in morphological characteristics and herbivore damage was largely attributable to phenotypic plasticity [48]. Knowing that *P. lanceolata* is a phenotypically plastic species with respect to morphological traits [49], it is well possible that all the variation we observed in *P. lanceolata* defence compounds along the plant diversity gradient is due to environmentally induced plasticity. Nevertheless, genetically determined differences have also been documented for *P. lanceolata* occurring in diverse habitat types [50]. It is thus conceivable that the differential abiotic and biotic conditions caused by plant species richness and functional group composition led to a genetic differentiation of *P. lanceolata* chemotypes in the experimental grassland plots of the Jena Experiment since their establishment in 2002.

### Conclusions

Plant species richness can both directly and indirectly modify the direct defence compounds of a common grassland species. Changes in light and nutrient availability and different levels of herbivore damage were partly responsible, but did not fully mediate plant diversity effects on direct chemical defence compounds in our study. These plant diversity effects on the chemical defence properties of individual plant species should be considered in the future as additional factors modulating plant-herbivore interactions in complex plant communities.

## Materials and Methods

### Study area and experimental design

The Jena Experiment, a large-scale biodiversity experiment, was established on former agricultural land in May 2002. The site was situated on the floodplain of the river Saale near to the city of Jena (Thuringia, Germany, 50°55′N, 11°35′E, 130 m a.s.l.). Mean annual air temperature in the area is 9.3°C and mean annual precipitation amounts to 587 mm [51]. Sixty experimental species typically occurring in Central European semi-natural mesophilic grasslands (Molinio-Arrhenatheretea [52]) were selected and classified into four functional groups: 16 grasses, 12 small herbs, 20 tall herbs, and 12 legumes (see [29] for details). The design of the Jena Experiment crosses the factors species richness (1, 2, 4, 8, 16 and 60 species) and plant functional group number (1, 2, 3 and 4 functional groups) near-orthogonally resulting in a total of 82 plots each 20×20 m in size. Species combinations for the experimental communities were determined by random draws with replacement. Initial sowing density was 1000 viable seeds per m^2^ in a substitutive design, where constant total density was obtained by reducing sowing densities per species proportional to the number of sown species per mixture. The experimental plots were arranged in four blocks parallel to the riverside to account for a gradient in soil texture. Plots were mown twice a year to mimic the management of extensive hay meadows (early June, September) and did not receive any fertilizer since sowing in 2002. The experimental grasslands were weeded twice per year (April, July) to maintain the sown species combinations.

### Study species

*Plantago lanceolata* (ribwort plantain) is a short-lived perennial rosette plant with adventitious roots that may reproduce by seeds or vegetatively forming new rosettes from axillary buds [53]. The species is self-incompatible and wind-pollinated. *Plantago lanceolata* was assigned to the functional group of “small herbs” in the Jena Experiment and belongs to the sown species combinations in 18 experimental plots covering the whole species-richness gradient (see Table S1 Supporting Information). Plantain produces the monoterpene-derived iridoid glycosides (IG), aucubin and catalpol, known to influence feeding by both specialist and generalist insect herbivores [24] as well as fungal pathogens growth [54]. Aucubin is the biosynthetic precursor to catalpol [55]. The latter is more heavily induced by herbivory than aucubin, especially by specialist herbivores [25].

### Plant sampling

In April 2008, when the vegetation was short enough to recognize plant individuals, density of *P. lanceolata* (number of individuals per m^2^) was determined in each plot on two to three randomly chosen 50×50 cm quadrats. In late May 2008, at estimated peak biomass shortly before first mowing, a transect excluding the outer 70 cm was installed perpendicular to the plot margin in each plot containing *P. lanceolata*. After every 50 cm the plant individual rooting closest to the point was chosen. One shoot ( = rosette with spirally arranged leaves) per plant individual was cut-off 2 cm above ground, put in a sealed plastic bag and transported in a cool box to the laboratory for further processing. In total, five reproductive and five vegetative shoots (from completely vegetative plant individuals) were harvested. From each shoot the two innermost adult leaves in the centre of the rosette ( = youngest fully expanded leaves = younger adult leaves) and three to four subsequent outer leaves ( = older adult leaves) were separated. Leaves of the younger and older adult cohort were spread separately on a board of white Plexiglas and covered with a non-reflecting Plexiglass plate. Leaves and a reference square (1.25×1.25 cm) were photographed from a fixed distance with the same resolution and without flash with a digital camera (Nikon Coolpix). Dry mass of leaf samples per cohort and residual shoot biomass were determined after drying for 48 h at 50°C. Digital photographs were analysed with the graphics software Adobe Photoshop 8.0. Firstly, total leaf area of the damaged leaves was determined using the pixel number of the reference square. Secondly, leaf parts missing due to herbivory were added to the digitized leaf by substituting consumed leaf area. Undamaged leaf parts were used as template when leaves were damaged along their margins. Afterwards, leaf area was determined again for the reconstructed leaves and leaf damage was quantified using the differences in pixel number between the reconstructed and the damaged leaves and converted into cm^2^ based on the reference square. Total area of damaged leaves and dry mass were used to calculate specific leaf area (SLA; mm^2^_leaf_ cm^−1^_leaf_).

Additionally, community leaf area index (LAI) was measured using a LAI-2000 Plant Canopy Analyzer (LI-COR, Lincoln, USA) to assess canopy density as an estimate of light availability at time of sampling. Five random measurements along a transect were taken to get a mean LAI value per plot. At the same time canopy height was determined averaging five individual measurements per plot.

### Chemical analyses

Dry material of the younger and older adult leaf cohort was ground to a fine powder with a ball mill. Approximately 10–20 mg (pooling replicates per plot separately for the younger and older leaf cohort of vegetative and reproductive shoots) were analysed for nitrogen concentrations (N_M_) with an elemental analyzer (Vario EL Element Analyzer, Hanau, Germany). For quantification of IG concentrations, 10 mg of ground leaf material (analyzing all leaf samples separately) were extracted with 1 ml 70% (v/v) methanol for 30 min at room temperature. After centrifugation at 4300× g for 10 min, 200 µl of the supernatant were diluted with 600 µl distilled water (1∶4). Samples were analyzed by High Performance Liquid Chromatography (HPLC) using an HP1100 System (Agilent Technologies, Waldbronn, Germany) equipped with a Nucleodur Sphinx RP column (Macherey-Nagel, 250×4.6 mm, 5 µm particle size) and applying a 0.05% trifluoroacetic acid (solvent A) –acetonitrile (solvent B) gradient at a flow rate of 1 ml min^−1^ at 25°C (injection volume 50 µl). Elution was accomplished with a gradient starting at 0% B, 0–10% B (10 min) followed by 10–40% B (10 min), 40–100% (0.1 min), 100% B (2 min), 100–0% B (0.1 min), and 0% B (4.9 min). The eluent was monitored by diode array detection between 190 and 360 nm (2 nm interval). Catalpol and aucubin were identified by comparing retention times and UV absorption spectra against commercial standards (catalpol, Wako Chemicals; aucubin, Carl Roth, Germany). Results are given as mg g_dw_^−1^ calculated from the peak areas at 200 nm using external calibration curves for catalpol and aucubin.

### Data analysis

Data were analysed with mixed-effects models using the *nlme* package of the statistical software R2.11.1 (R Development Core Team, http://www.R-project.org). If necessary, data were log-transformed to meet the assumptions of normality and homogeneity of variances. Block, plot identity and shoot identity were entered in a nested sequence as random effects for analyses of data recorded on older and younger adult leaves of reproductive and vegetative shoots. The block was entered to remove variance caused by the gradient of edaphic conditions in the field site, but also effects of the block-wise organized management (weeding, mowing) and data collection. Plot identity accounts for replicated sampling of shoots per plot, while shoot identity accounts for older and younger adult leaves originating from the same shoot. Starting from a constant null model the fixed effects were entered in the following sequence: species richness as log-linear term (SR), functional group number as linear term (FG), life stage as contrast vegetative vs. reproductive shoots (stage) and its interactions with the previous terms (“stage × SR”, “stage × FG”), leaf age as contrast younger vs. older leaf cohort and its interaction with life stage. In alternative models, a contrast for the presence/absence of legumes and grasses respectively characterising plant composition of the experimental plots and its interaction with life stage were fitted. The maximum likelihood method was applied and likelihood ratio (L) tests were used to assess the statistical significance of model improvement. The same principles were applied to test effects of plant diversity on density (with block as random effect) and shoot biomass (with plot identity nested in block as random effects) of *P. lanceolata*.

To test whether effects of plant diversity were mediated indirectly through changes in resource availability and herbivory along the plant diversity gradient leaf damage, SLA and N_M_ were implemented as covariates in mixed-effects model analyses of total IG, aucubin and catalpol concentrations. To determine their fitting order, firstly each covariate was fitted in a separate model. The Akaike's information criterion (AIC, [56]) was used for model comparison, where smaller values of AIC indicate higher predictive power of the respective statistical model. The model with the lowest AIC containing one of the covariates was selected, and in a further step the respective model was extended fitting additionally one of the remaining covariates in alternative models. Likelihood ratio tests were used to test whether an addition of terms led to a significant model improvement. The full set of covariates including non-significant terms was fitted before the experimental factors to test whether covariates could explain a significant proportion of variation ascribed to treatment effects.

Finally, data were averaged at the plot level across both leaf cohorts of vegetative and reproductive shoots. Simple path analyses based on a correlation matrix of standardized variables [57] was performed with Amos 5 (http://amosdevelopment.com) to evaluate the relative contribution of leaf damage, SLA and N_M_ as predictor variables to explain variation in IG concentrations without accounting for within-plot variation.

## Supporting Information

Figure S1 Relationships between species richness and plant density and shoot biomass of *Plantago lanceolata*. Plant density counted in each plot corrected for sown proportions (i.e. multiplied by the number of sown species to account for the substitutive distribution of seed numbers per species dependent on the number of sown species) plotted against sown species richness (a), shoot biomass as means across reproductive and vegetative shoots of *P. lanceolata* per plot plotted against sown species richness (b), and as means (± SE) for reproductive (rep) and vegetative (veg) shoots across plots (c).
(TIF)

Table S1 Details of the 18 experimental plots containing *Plantago lanceolata* including sown species richness (SR), number of functional groups (FG), functional group composition (FG-Id; Grasses = G, Legumes = L, Small herbs = S, Tall herbs = T) and species identity (Sp-Id).
(DOCX)
