# Supplementary material for: Plant Community Diversity Influences Allocation to Direct Chemical Defence in Plantago lanceolata
Source: PLoS One. 2011 Dec 9;6(12):e28055. doi: 10.1371/journal.pone.0028055 (PMC3235097; doi:10.1371/journal.pone.0028055)
Supplement: Table S1 — Details of the 18 experimental plots containing Plantago lanceolata including sown species richness (SR), number of functional groups (FG), functional group composition (FG-Id; Grasses = G, Legumes = L, Small herbs = S, Tall herbs = T) and species identity (Sp-Id). (DOCX) [file pone.0028055.s002.docx]

**Table S1**: Details of the 18 experimental plots containing *Plantago lanceolata* including sown species richness (SR), number of functional groups (FG), functional group composition (FG-Id; Grasses = G, Legumes = L, Small herbs = S, Tall herbs = T) and species identity (Sp-Id).

| SR | FG | FG-Id | Sp-Id | |  |  |  |  |  |  |  |  |  |  |  |  |  |  |
| --- | --- | --- | --- | --- | --- | --- | --- | --- | --- | --- | --- | --- | --- | --- | --- | --- | --- | --- |
| 1 | 1 | S | Pl |  |  |  |  |  |  |  |  |  |  |  |  |  |  |  |
| 2 | 1 | S | Pl | Bp |  |  |  |  |  |  |  |  |  |  |  |  |  |  |
| 2 | 2 | SG | Pl | **Pp** |  |  |  |  |  |  |  |  |  |  |  |  |  |  |
| 2 | 2 | SL | Pl | *Td* |  |  |  |  |  |  |  |  |  |  |  |  |  |  |
| 4 | 1 | S | Pl | Ar | Pu | Pv |  |  |  |  |  |  |  |  |  |  |  |  |
| 4 | 2 | SG | Pl | Pu | **Be** | **Pt** |  |  |  |  |  |  |  |  |  |  |  |  |
| 4 | 2 | SL | Pl | To | *Lp* | *Ml* |  |  |  |  |  |  |  |  |  |  |  |  |
| 4 | 4 | SGLT | Pl | **Ae** | *Tc* | As |  |  |  |  |  |  |  |  |  |  |  |  |
| 4 | 4 | SGLT | Pl | **Fp** | *Ov* | Cp |  |  |  |  |  |  |  |  |  |  |  |  |
| 8 | 2 | SG | Pl | Ar | To | Vc | **Ao** | **Av** | **Bh** | **Fr** |  |  |  |  |  |  |  |  |
| 8 | 4 | SGLT | Pl | Lh | **Lu** | **Ti** | *Tc* | *Tf* | As | Dc |  |  |  |  |  |  |  |  |
| 16 | 2 | SG | Pl | Bp | La | Lh | Pu | Pv | Rr | Vc | **Ae** | **Ao** | **Ap** | Be | **Fp** | **Fr** | **Hl** | **Ph** |
| 16 | 3 | SLT | Pl | Ar | Bp | La | Lh | Vc | *Lc* | *Mv* | *Ov* | *Th* | *Tr* | Am | Gp | Ka | Ra | So |
| 16 | 4 | SGLT | Pl | Ar | Rr | To | **Ao** | **Av** | **Bh** | **Pp** | *Lp* | *Lc* | *Tc* | *Vc* | As | Cc | Gp | Ta |
| 60 | 4 | SGLT | Pl | all species | | |  |  |  |  |  |  |  |  |  |  |  |  |
| 60 | 4 | SGLT | Pl | all species | | |  |  |  |  |  |  |  |  |  |  |  |  |
| 60 | 4 | SGLT | Pl | all species | | |  |  |  |  |  |  |  |  |  |  |  |  |
| 60 | 4 | SGLT | Pl | all species | | |  |  |  |  |  |  |  |  |  |  |  |  |

Complete species list and abbreviations of species names:

***Small herbs:***

*Ajuga reptans* L. (Lamiaceae) = Ar; *Bellis perennis* L. (Asteraceae) = Bp; *Glechoma hederacea* L. (Lamiaceae); *Leontodon autumnalis* L. (Asteraceae) = La; *Leontodon hispidus* L. (Asteraceae) = Lh; *Plantago lanceolata* L. (Plantaginaceae) = Pl; *Plantago media* L. (Plantaginaceae); *Primula veris* L. (Primulaceae) = Pv; *Prunella vulgaris* L. (Lamiaceae) = Pu; *Ranunculus repens* L. (Ranunculaceae) = Rr; *Taraxacum officinale* Wiggers (Asteraceae) = To; *Veronica chamaedrys* L. (Scrophulariaceae) = Vc

***Grasses:***

*Alopecurus pratensis* L. (Poaceae); *Anthoxanthum odoratum* L. (Poaceae) = **Ao**; *Arrhenatherum elatius* (L.) J. et C. Presl (Poaceae) = **Ae**; *Avenula pubescens* (Huds.) Dum. (Poaceae) = **Av**; *Bromus erectus* Huds. (Poaceae) = **Be**; *Bromus hordeaceus* L. (Poaceae) = **Bh**; *Cynosurus cristatus* L. (Poaceae); *Dactylis glomerata* L. (Poaceae); *Festuca pratensis* Huds. (Poaceae) = **Fp**; *Festuca rubra* L. (Poaceae) = **Fr**; *Holcus lanatus* L. (Poaceae) = **Hl**; *Luzula campestris* (L.) Dc. (Juncaceae) = **Lu**; *Phleum pratense* L. (Poaceae) = **Ph**; *Poa pratensis* L. (Poaceae) = **Pp**; *Poa trivialis* L. (Poaceae) = **Pt**; *Trisetum flavescens* (L.) P. Beauv. (Poaceae) = **Ti**

***Legumes:***

*Lathyrus pratensis* L. (Fabaceae) = *Lp*; *Lotus corniculatus* L. (Fabaceae) = *Lc*; *Medicago lupulina* L. (Fabaceae) = *Ml*; *Medicago x varia* Martyn (Fabaceae) = *Mv*; *Onobrychis viciifolia* Scop. (Fabaceae) = *Ov*; *Trifolium campestre* Schreb. (Fabaceae) = *Tc*; *Trifolium dubium* Sibth. (Fabaceae) = *Td*; *Trifolium fragiferum* L. (Fabaceae) = Tf; *Trifolium hybridum* L. (Fabaceae) = Th; *Trifolium pratense* L. (Fabaceae); *Trifolium repens* L. (Fabaceae) = *Tr*; *Vicia cracca* L. (Fabaceae) = *Vc*

***Tall herbs:***

*Achillea millefolium* L. (Asteraceae) = Am; *Anthriscus sylvestris* (L.) Hoffm. (Apiaceae) = As; *Campanula patula* L. (Campanulaceae) = Cp; *Cardamine pratensis* L. (Brassicaceae); *Carum carvi* L. (Apiaceae) = Cc; *Centaurea jacea* L. (Asteraceae); *Cirsium oleraceum* (L.) Scop. (Asteraceae); *Crepis biennis* L. (Asteraceae); *Daucus carota* L. (Apiaceae) = Dc; *Galium album* Mill. (Rubiaceae); *Geranium pratense* L. (Geraniaceae) = Gp; *Heracleum sphondylium* L. (Apiaceae); *Knautia arvensis* (L.) J.M. Coult. (Dipsacaceae) = Ka; *Leucanthemum vulgare* Lam. (Asteraceae); *Pastinaca sativa* L. (Apiaceae); *Pimpinella major* (L.) Huds. (Apiaceae); *Ranunculus acris* L. (Ranunculaceae) = Ra; *Rumex acetosa* L. (Polygonaceae); *Sanguisorba officinalis* L. (Rosaceae) = So; *Tragopogon pratensis* L. (Asteraceae) = Ta
